# Macaque mothers’ responses to the deaths of their infants

**DOI:** 10.1098/rsbl.2024.0484

**Published:** 2025-04-16

**Authors:** Emily A. Johnson, Flóra Talyigás, Alecia Carter

**Affiliations:** ^1^ Department of Anthropology, University College London, London, UK; ^2^ Department of Genetics, Ecology and Evolution, University College London, London, UK

**Keywords:** comparative thanatology, grief, *Macaca mulatta*, macaque, primate

## Abstract

Although it is understood that all humans grieve the death of close social partners, little empirical research has addressed animals’ responses to death. In this study, we collected quantitative data on the behaviour of 11 bereaved rhesus macaque (*Macaca mulatta*) mothers at Cayo Santiago to the natural deaths of their infants and matched, non-bereaved controls. Our research focused on behavioural signs of grief, including loss of appetite, lethargy, increased stress and social withdrawal, highlighting that such responses are documented in the human literature, but could be found in mammalian taxa. Using mixed models, we found that, contrary to prediction, bereaved mothers spent less time resting than the non-bereaved control females in the first two weeks after their infants’ deaths. There were no other behavioural markers of grief. We conclude that mothers showed a short-term behavioural response to their bereavement that does not match human’s prolonged ‘despair’ grief. We propose that mothers’ behavioural responses might be a form of ‘protest’ grief, as is seen in primate infants when separated from mothers and in humans, or do not grieve. We hope to advance the field of comparative thanatology by providing a framework and novel predictions for future studies in this area.

## 1. Introduction

Grief is generally accepted as a universal human behaviour, despite diverse cultural mourning practices [1]. While there is no precise definition of grief, grief is understood as a negative emotional response to (perceived) loss or regret [2]. Throughout this article, we consider grief to be distinct from bereavement. Bereavement describes the state of losing someone to death but does not imply the negative emotional or behavioural response of grief [3]. Although cultural practices can exacerbate some symptoms of grief [2] by, for example, proscribing social interaction [4], humans’ behavioural changes in response to grief commonly include: reduced appetite [5]; lethargy due to sleep disturbances [6]; increased anxiety [7]; and social isolation [8,9]. Given that these behavioural responses to grief could be interpreted as maladaptive, particularly if they are prolonged, the evolution of grief presents an evolutionary puzzle. Two hypotheses have been proposed to explain its evolution: the byproduct and adaptive hypotheses. The byproduct hypothesis suggests that grief evolved as a negative byproduct of strong selection on enduring bonds in social animals. Bonds are maintained in life by feelings of panic and pain if there is physical separation; an individual’s death results in irreparable separation and prolonged panic/pain [10,11]. The adaptive hypothesis suggests grief was selected, at least in humans, because it is an honest signal of socially desirable traits—commitment and loyalty—of the bereaved [12].

Studies of animal grief are generally limited to anecdotal reports of limited numbers of events [13]. However, some studies have used secondary analysis to explore physiological and behavioural responses to bereavement that could provide evidence for grief. Wild female chacma baboons (*Papio ursinus*) who lost a close relative to predation had a greater change in their faecal glucocorticoids in the month after the loss compared to control individuals [14]. Similarly, bereaved humans who are grieving secrete higher levels of cortisol [15,16]. Behavioural evidence for animal grief could come from observations of mothers’ behaviour after the deaths of their infants. A retrospective study quantified the behaviours of 18 bonnet macaque (*Macaca radiata*) mothers before and after the deaths of their infants and juveniles [17]. Mothers performed more self-directed behaviours, an indicator of acute stress, and vocalized more after their infants’ deaths. These behaviours are similar to human grief behaviour [17], and could indicate a conserved response in primates. However, these data were retrospective and compared females when they did and did not have an infant; it is not possible to determine whether the change in behaviour was due to grief or an external environmental or other social variable [18]. Similarly, elephants (*Loxodonta africana*) who visit conspecific carcasses may show self-directed behaviours and temporal gland streaming, which may indicate an emotional response [19], but we are not aware of data showing that this occurs away from the carcass, which would indicate a grief response rather than a response to the stimulus of the carcass. Finally, there is some evidence that companion animals’ behaviour shows grief-like changes after a co-residing conspecific has died, including less time playing, more time resting, and diminished appetite [20,21], but these survey data are influenced by the owners’ perceptions and do not account for the change in the social environment of the bereaved individual. We know of no studies that have set out to collected data on animals’ responses to bereavement.

In this study, we aimed to identify potential behavioural indicators of grief in bereaved rhesus macaque mothers at the Caribbean Primate Research Center (CPRC) in Cayo Santiago. We worked from the hypothesis that grief is an evolutionarily conserved emotional response to bereavement [22,23]. We focused our data collection and predictions on behavioural markers of grief in humans including: lethargy, lack of appetite, increased stress and decreased socialization. Based on this literature, we predicted that, compared to control females, bereaved mothers would: (i) spend more time resting; (ii) spend less time feeding; (iii) perform more behavioural indicators of acute stress; and (iv) spend less time grooming and being groomed. Self-directed displacement behaviours are associated with stress and anxiety [24]. In humans, grief is associated with increased anxiety [7], and bereavement is defined as a stressful event. Therefore, we expected displacement behaviours to increase in the bereaved mothers indicative of a grief response.

## 2. Material and methods

### (a) Study site and groups

Fieldwork was conducted on the free-ranging colony of rhesus macaques in Cayo Santiago (18.1564° N, 65.7338° W) between May and September 2022. The population is maintained by the CPRC through the provision of water and monkey chow, with the macaques also feeding on natural vegetation on the island. This colony comprised 1497 free-ranging macaques across 13 troops (May 2022). Rhesus macaques are seasonal breeders, with a three month annual birthing season where 72% of births occur [25]. The study was timed to occur during the peak of births. All corpses on Cayo Santiago are removed by CPRC staff when they are found.

### (b) Focal observations

To quantify mothers’ responses to the deaths of their infants, we conducted focal observations on all mothers who lost an infant (hereafter referred to as bereaved mothers; *n* = 11 bereaved mothers) and matched controls for each bereaved mother (*n* = 11 control individuals). Because previous behavioural research has been conducted retrospectively on bereaved mothers in non-human primates (hereafter referred to as ‘primates’), we were uncertain about the best-matched control. We thus chose two unrelated, similarly aged control females from the same group: a female who (i) currently had an infant under the age of one and another who (ii) did not have an infant under 1 year. The first control was chosen in case the bereaved mother carried the corpse of her infant and could thus interact with it. However, most (*n* = 9, 82%) bereaved females did not carry their infants’ corpses. In two cases, females did carry the corpse, but this was for <2 days. We decided to remove the control with an infant because differences between the behaviour of the bereaved and control could arise not because of grief in the bereaved individual, but because mothers have different energetic requirements to non-mothers [26], and affiliative behaviour directed to mothers from others differs as a result of primates’ natal attraction [27]. Instead, we included only the second control: an unrelated, similarly aged female without an infant (*n* = 11). We avoided heavily pregnant females as controls as a birth during the data collection period would invalidate them.

We recorded potential behaviours that could be considered analogous to those associated with human grief using focal observations of 20 min duration. The data were collected daily on weekdays between 07.30 and 14.00 (AST) when weather permitted travel to the island. Observations were made by E.A.J. or F.T. E.A.J. trained F.T. in data collection and conducted focal observations together until their data collection matched (>95% agreement) for three observations. Data were collected opportunistically but we prioritized searching for individuals with the lowest number of observations in the first instance, and where researcher availability was limited because deaths had occurred in different troops, we prioritized observing bereaved females as close to the deaths as possible. The median number of days since the infants’ deaths that females were observed was 16 (IQR = 7–32) (bereaved median and IQR = 13 days, 5−27; control: 14, 7−34). We recorded durations of behavioural states during the focal observations, which included: feeding, grooming, travelling, travelling while feeding, resting and displacement behaviours. Displacement behaviours included agitated locomotion, pacing and the following self-directed behaviours: self-grooming, self-scratching, self-touch and body shake (see electronic supplementary material, table 1, for definitions). The behavioural observations were recorded on smartphones using the Cybertracker software (cybertracker.org). If the focal individual moved out of sight for more than 5 min, data collection was terminated and that observation discarded, and wherever possible, a new focal observation was started when the focal individual had been re-identified. If more than one focal observation was made on an individual in 1 day, we waited at least an hour to ensure the observations were independent.

Given the short observation time that observers had in the field (three months), the large sizes of the study troops, and the limited number of observers for this project, we were not able to learn all troop members’ identities or collect sufficient data to determine macaques’ absolute ranks. Instead, we estimated focal individuals’ relative positioning (low, middle and high) in each troop’s dominance hierarchy based on individuals’ access to provisioned food, and dominance and submissive behaviour during provisioning.

### (c) Statistical analyses

All analyses were conducted in R v. 3.3.3. Our response variables were the durations (seconds) spent in each of the four behaviours of interest: time spent resting, feeding, displacement behaviours and grooming. We are aware that, because these are mutually exclusive categories, these response variables are not independent of one another and are cautious in our interpretation of our findings because of this (see below). Additionally, while the durations are discrete counts, the means were large and approached a normal distribution in most cases. Because of this, we analysed these data using a normal distribution with the duration of the behaviour as the response and the total duration of each observation as an offset. We checked that the residuals were normally distributed and quantile–quantile plots were linear. We also included in all models the following control variables as covariates: (i) the females’ age (years) to control for age differences in behaviour [28]; (ii) the females’ rank category (low, middle and high) to control for rank differences in, e.g., access to grooming; and (iii) the hour of the day, to control for temporal differences in behaviour across the day. All control variables were retained; no model simplification was done. Because our data contained repeated measures, we conducted linear mixed models (LMM) with individual identity nested in the pair (a value indicating the bereaved female and the control) as a random effect. In addition to the model residual checks, we also checked for multicollinearity using the function *vif* in the *car* R package [29]. All variance inflation factors were <4, except those involved in the interaction in those models that had them, which is expected. Because lme4 does not report *p*-values for LMMs, we considered variables as significant if the *t*-value was >|1.94|. We also made null model comparisons using ANOVA, comparing the full model to a ‘null’ model that did not include the variable of interest.

In all models, the predictor of interest was the females’ status (i.e. bereaved or control). It is likely that bereaved mothers’ behaviour returns to baseline levels after time [14]. We thus investigated the role of time since the infants’ deaths in two ways. First, we included the interaction between mothers’ status and the time since the infants’ death (days) after each infant’s death. If this interaction was not significant, we removed the interaction to simplify the model and kept the number of days since the infant’s death as a covariate. Second, because the change in behaviour over time could be nonlinear, we also tested whether the behaviour of the bereaved mother differed from that of the control mother without an infant at differing time intervals: within two weeks, within three weeks and within one month of the infants’ deaths. As a result, for each response variable, we ran five ANOVA comparisons: one to determine whether the interaction between days since death and female status had predictive power, and four models to determine the predictive power of the females’ status (bereaved or control) across the four subsets of the sample: full, one month, three weeks and two weeks.

## 3. Results

Data were collected on 22 individuals over 93 days representing 11 deaths across seven troops. Infants were on average (median) 9 days old at their death (range = 0–17 days). One infant was recorded as a stillbirth, one infant was found dead, three infants went missing and the remaining six infants were seen dead with their mother on the day of death. Depending on the timescale of the analysis, 95−184 focal observations were collected (*N*_full_ = 184, *N*_month_ = 136, *N*_three weeks_ = 119, *N*_two weeks_ = 95) for a total of 2528 min of observation (bereaved total = 1437 min, control = 1091 min) with, on average (median), nine observations per individual (range = 6–12 observations) for a duration of 158.7 min (median) per individual (range = 114.24–222.2 min).

**Figure 1.**
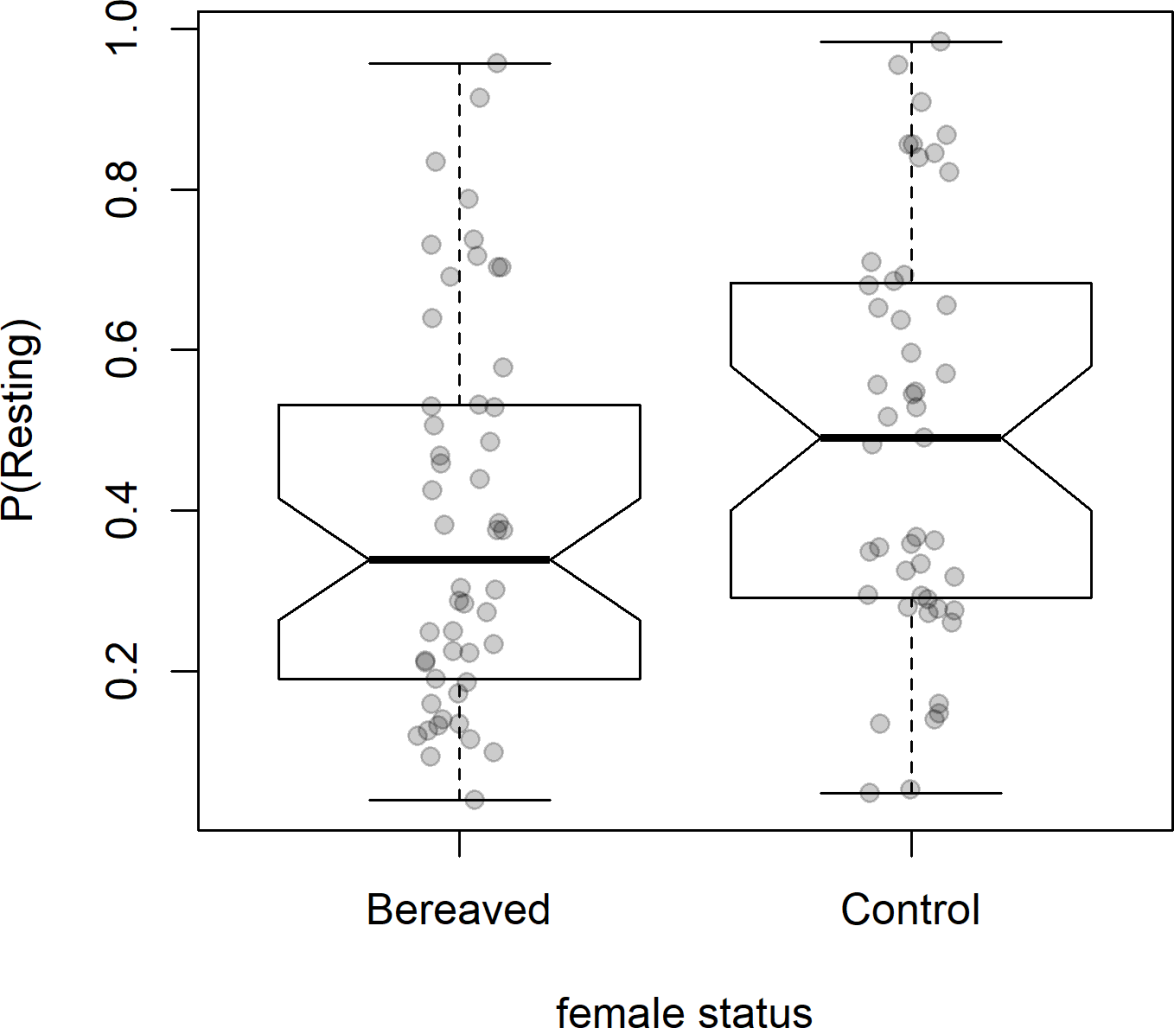
Box-plot of time spent resting in the two weeks following an infant’s death in bereaved and control females.

We found little support for our hypotheses: there was no effect of the females’ status (control or bereaved) on the time spent foraging, grooming or doing displacement behaviours at any timescale (see electronic supplementary material, table 2, for *χ*^2^ tests and electronic supplementary material, table 3, for a full list of the models’ estimates). However, we did find some evidence for a short-term effect of bereavement on resting: contrary to our prediction, bereaved females spent *less* time resting in the first two weeks after their infants’ deaths compared to control females (table 1 and figure 1).

**Table 1.** Summary of the model for time spent resting in the first two weeks after an infant’s death.

predictor	*β*	s.e.	*t*
intercept	−780.82	260.44	−3.00
status: control^a^	144.47	64.52	2.24
days	1.32	5.97	0.22
age	7.19	8.74	0.82
rank: low^b^	−130.44	108.98	−1.20
rank: middle^b^	−192.2	111.36	−1.73
hour	15.27	19.80	0.77

Shown are the model outputs for the two weeks resting model, showing the fixed effects (predictor), the estimates (*β*), standard errors (s.e.) and *t*-value (*t*). a: reference cartegory = bereaved, b: reference category = high rank.

Shown are the raw proportions of time spent resting in each observation (*n* = 95) in the first two weeks after an infant’s death according to a female’s status (bereaved (*n* = 11) and control females without an infant (*n* = 11)). N.B. the proportions of time spent resting are shown for ease of interpretation; however, the models analysed total time spent in the behaviour, with observation time as an offset.

## 4. Discussion

We aimed to conduct what we believe to be the first systematic study that set out to test whether non-human primate mothers showed similar behavioural responses to the deaths of their infants as is seen in human grief. We tested for evidence of behavioural markers of grief in 11 bereaved mothers compared to matched control females with no infants over the same period. Contrary to our predictions based on the human literature, we found that bereaved mothers rested less, not more, in the first two weeks after their infants’ deaths. We found no other evidence for behavioural differences between the bereaved and control individuals. Two aspects of this finding are surprising: first, that the time window was relatively short, and second, that females did not show evidence of lethargy, as has been shown in humans. Regarding the first aspect, it is possible that social primates use coping mechanisms to return to baseline levels relatively quickly after a bereavement. For example, chacma baboons who suffered the loss of a close relative also showed a short-term (one month) increase in glucocorticoids compared to control females [30]. It is not clear whether the physiological response to the bereavement was shorter than one month, but it is possible that primates can, on average, return to baseline levels more rapidly than humans. Mother–infant separation studies also support a relatively short (i.e. several days) period of maternal ‘emotional disturbance’ after separation from their infant, at least when still in visual contact with their infant [31].

The second aspect—lower time spent resting after bereavement—may highlight the simplistic and insufficient nature of our original predictions. Mother–infant separation studies in primates describe two stages of response to separation: both mothers’ (reviewed in [32]) and infants’ responses (reviewed in [33,34]) to separation include an initial period of ‘agitation’, marked by higher levels of locomotion and vocalization (termed ‘protest’), followed by, in infants, a period of decreased locomotion and activity (termed ‘despair’: [28]). Primate mothers in captivity, in contrast to infants, seem not to enter the ‘despair’ stage after the initial ‘protest’ stage of separation in mother–infant separation studies [35,36]. Similarly, adult female chacma baboons showed a physiological response to bereavement, but no behavioural markers of despair grief: indeed, females *increased* their social networks in the month after a close relative’s death [30], rather than showing less engagement with others. It may be that the bereaved mothers in this study rested less because they were in the ‘protest’ stage of separation from their infant, and were more active, though we are clear that we did not specifically test whether the females were more active. We propose more nuanced predictions for comparative thanatological studies in line with the biphasic protest–despair stages of grief described in separation studies in mammals. Moving forward, we would predict the following behavioural differences in bereaved individuals compared to matched controls: (i) an initial period of protest grief characterized by an increase in behavioural state changes (as an indicator of agitation), activity, and separation vocalizations. (ii) This stage may or may not be followed by a subsequent period of despair grief, indicated by three of the symptoms of grief with which we started (§1): an increase in lethargy, lack of appetite, and decreased socialization. The second stage is more complicated to test because it is unlikely that all individuals will cross from protest into despair grief [11]. It is not yet understood what makes some individuals more susceptible to crossing over from protest to despair grief, but it is possible that the initial protest stage is relatively short (several days to one month).

There are at least three alternative explanations to the biphasic grief hypothesis we suggest above for why we found little support for our predictions in this sample. In the first case, it is possible that macaque mothers do not experience grief after the death of their infant. Neurological evidence suggests that the emotional system responsible for protest grief is basal in mammals [23] and that primates can suffer from depression-like symptoms in response to adverse events [37,38]. Indeed, separation from peers has been used to induce depression in primates [37]. However, few observations of primate mothers suggest that they suffer from despair grief when separated from their offspring (but see [17]): observations comparing captive rhesus macaque mothers after their infants’ removal to mothers whose infants were not removed showed no differences in behaviour and hair cortisol [39]. Additionally, there was no change in zoo-housed chimpanzee (*Pan troglodytes*) mothers’ behaviour after their juvenile offspring were removed [35]. Taken together with the findings of this study, it is possible that non-human primate mothers do not grieve after their infants’ deaths, at least not in a manner homologous to human grief.

A second alternative hypothesis regards the sample. Our small sample size may have limited the power we had to detect an effect. It is possible that with a larger sample, ‘trends’ in the data that were apparent with the small sample (e.g. lower grooming in bereaved mothers in the two weeks after an infant’s death; electronic supplementary material, table 1) could be more apparent. Additionally, our observational sample included mothers who lost very young infants, including a stillbirth, with the oldest infant being 17 days old. Mothers of very young infants may not show strong responses to their infants’ deaths because they have not yet formed a strong mother–infant bond. Data from dairy cattle may support this hypothesis: mothers whose calves were removed within 24 h of birth had lower behavioural responses to the separation than when older calves were removed (4–14 days) (reviewed in [40]). However, this effect is likely to be restricted to the few days postpartum: maternal motivation in primates is heightened in the early postpartum period [41] and dairy cattle respond strongly to their calves’ removal after 4 days [40]. Additionally, primate mothers carry their infants’ corpses for longer durations when the infant dies at a younger age, which may suggest that mothers are more strongly bonded to younger infants [42]. Taken together, these cross-species findings could suggest that the deaths of infants in the first days postpartum could result in a diminished or no grief response.

Finally, our third alternative hypothesis regards noise, i.e. between-individual differences in response to bereavement. Part of the difficulty of studying grief is that it is highly individualized. Some generalizations can be made from the human literature, but even human grief is understudied in a cross-cultural context [2,43], and the full diversity of responses are unknown. Similar diversity in responses is apparent in primates; for example, there is high variation in the duration that mothers’ carry their infants’ corpses [42], and in three bonnet macaque cases, behavioural change remained up to three months after an infant’s death [17]. Future studies could attempt to predict individuals’ responses to bereavement using a combination of physiological and behavioural data.

We aimed for this to be the first systematic study that set out to quantify maternal behavioural responses to bereavement in response to natural deaths. Although our findings did not provide support for our original predictions, our interpretations provide new, testable predictions. We emphasize the need for clarity around protest and despair grief in mammals and suggest the field of comparative thanatology could consider these as different processes. We encourage other systematic data collection on primates’ responses to bereavement.

## Data Availability

The data that support the findings of this study are openly available from Zenodo at [44]. Electronic supplementary material is available online [45].

## References

[1] Bui E. 2018 Grief: From normal to pathological reactions. In Clinical handbook of bereavement and grief reactions (ed. E Bui), pp. 85–101. (Current Clinical Psychiatry).Totowa, NJ: Humana Press.

[2] Rosenblatt PC. 2008 Grief across cultures: a review and research agenda. In Handbook of bereavement research and practice: advances in theory and intervention (eds MS Stroebe, RO Hansson, H Schut, W Stroebe), pp. 207–222. Washington, DC: American Psychological Association.

[3] Zisook S, Shear K. 2009 Grief and bereavement: what psychiatrists need to know. World Psychiatry **8**, 67–74. (10.1002/j.2051-5545.2009.tb00217.x) 19516922 PMC2691160

[4] Rosenblatt PC, Nkosi BC. 2007 South African Zulu widows in a time of poverty and social change. Death Stud. **31**, 67–85. (10.1080/07481180600995214) 17131562

[5] Fried EI *et al*. 2015 From loss to loneliness: the relationship between bereavement and depressive symptoms. J. Abnorm. Psychol. **124**, 256–265. (10.1037/abn0000028) 25730514

[6] Lancel M, Stroebe M, Eisma MC. 2020 Sleep disturbances in bereavement: a systematic review. Sleep Med. Rev. **53**, 101331. (10.1016/j.smrv.2020.101331) 32505968

[7] Zisook S, Schneider D, Shuchter SR. 1990 Anxiety and bereavement. Psychiatr. Med. **8**, 83–96. 2185504

[8] Breen LJ, O’Connor M. 2011 Family and social networks after bereavement: experiences of support, change and isolation. J. Fam. Ther. **33**, 98–120. (10.1111/j.1467-6427.2010.00495.x)

[9] Rokach A. 1990 Surviving and coping with loneliness. J. Psychol. **124**, 39–54. (10.1080/00223980.1990.10543204) 2319485

[10] Nesse RM. 2005 An evolutionary framework for understanding grief. In Spousal bereavement in late life (eds D Carr, RM Nesse, CB Wortman), pp. 195–226. New York, NY: Springer Publishing Company.

[11] Panksepp J, Watt D. 2011 Why does depression hurt? Ancestral primary-process separation-distress (PANIC/GRIEF) and diminished brain reward (SEEKING) processes in the genesis of depressive affect. Psychiatry **74**, 5–13. (10.1521/psyc.2011.74.1.5) 21463165

[12] Winegard BM, Reynolds T, Baumeister RF, Winegard B, Maner JK. 2014 Grief functions as an honest indicator of commitment. Personal. Soc. Psychol. Rev. **18**, 168–186. (10.1177/1088868314521016) 24501093

[13] King B. 2013 How animals grieve. Chicago, IL: University of Chicago Press.

[14] Engh AL, Beehner JC, Bergman TJ, Whitten PL, Hoffmeier RR, Seyfarth RM, Cheney DL. 2005 Behavioural and hormonal responses to predation in female chacma baboons (Papio hamadryas ursinus). Proc. R. Soc. B **273**, 707–712. (10.1098/rspb.2005.3378) PMC1560071 16608690

[15] Buckley T, Sunari D, Marshall A, Bartrop R, McKinley S, Tofler G. 2012 Physiological correlates of bereavement and the impact of bereavement interventions. Dialogues Clin. Neurosci. **14**, 129–139. (10.31887/dcns.2012.14.2/tbuckley) 22754285 PMC3384441

[16] Richardson VE, Bennett KM, Carr D, Gallagher S, Kim J, Fields N. 2015 How does bereavement get under the skin? The effects of late-life spousal loss on cortisol levels. J. Gerontol. Ser. B **70**, 341–347. (10.1093/geronb/gbt116) 24259378

[17] Arlet ME, Anand A, Saikia A, Kaasik A, Sirigeri S, Isbell LA, Singh M. 2023 Behavior of mothers after infant loss in bonnet macaques (Macaca radiata). Int. J. Primatol. **44**, 1182–1199. (10.1007/s10764-023-00395-2)

[18] Carter A, Huchard E. 2024 What is the ‘least bad’ control in comparative thanatology studies? A comment on Arlet et al., 2023. Int. J. Primatol. **45**, 1001–1003. (10.1007/s10764-023-00411-5)

[19] Goldenberg SZ, Wittemyer G. 2020 Elephant behavior toward the dead: a review and insights from field observations. Primates **61**, 119–128. (10.1007/s10329-019-00766-5) 31713106

[20] Greene B, Vonk J. 2024 Is companion animal loss cat-astrophic? Responses of domestic cats to the loss of another companion animal. Appl. Anim. Behav. Sci. **277**, 106355. (10.1016/j.applanim.2024.106355)

[21] Uccheddu S *et al*. 2022 Domestic dogs (Canis familiaris) grieve over the loss of a conspecific. Sci. Rep. **12**, 1920. (10.1038/s41598-022-05669-y) 35210440 PMC8873218

[22] de Waal FBM. 2011 What is an animal emotion? Ann. NY Acad. Sci. **1224**, 191–206. (10.1111/j.1749-6632.2010.05912.x) 21486301

[23] Panksepp J, Watt D. 2011 What is basic about basic emotions? Lasting lessons from affective neuroscience. Emot. Rev. **3**, 387–396. (10.1177/1754073911410741)

[24] Maestripieri D, Schino G, Aureli F, Troisi A. 1992 A modest proposal: displacement activities as an indicator of emotions in primates. Anim. Behav. **44**, 967–979. (10.1016/s0003-3472(05)80592-5)

[25] Hernandez‐Pacheco R, Delgado DL, Rawlins RG, Kessler MJ, Ruiz‐Lambides AV, Maldonado E, Sabat AM. 2016 Managing the Cayo Santiago rhesus macaque population: the role of density. Am. J. Primatol. **78**, 167–181. (10.1002/ajp.22375) 25597512 PMC4504838

[26] Emery Thompson M. 2013 Comparative reproductive energetics of human and nonhuman primates. Annu. Rev. Anthropol. **42**, 287–304. (10.1146/annurev-anthro-092412-155530)

[27] Silk JB. 1999 Why are infants so attractive to others? The form and function of infant handling in bonnet macaques. Anim. Behav. **57**, 1021–1032. (10.1006/anbe.1998.1065) 10328788

[28] Kato E. 1999 Effects of age, dominance, and seasonal changes on proximity relationships in female Japanese macaques (Macaca fuscata) in a free-ranging group at Katsuyama. Primates **40**, 291–300. (10.1007/bf02557553)

[29] Fox J *et al*. 2012 car: Companion to Applied Regression. See https://CRAN.R-project.org/package=car.

[30] Engh AL, Beehner JC, Bergman TJ, Whitten PL, Hoffmeier RR, Seyfarth RM, Cheney DL. 2006 Behavioural and hormonal responses to predation in female chacma baboons (Papio hamadryas ursinus). Proc. R. Soc. B **273**, 707–712. (10.1098/rspb.2005.3378) PMC1560071 16608690

[31] Seay B, Hansen E, Harlow HF. 1962 Mother–infant separation in monkeys. J. Child Psychol. Psychiatry. **3**, 123–132. (10.1111/j.1469-7610.1962.tb02047.x) 13987549

[32] Maestripieri D. 2011 Emotions, stress, and maternal motivation in primates. Am. J. Primatol. **73**, 516–529. (10.1002/ajp.20882) 20872879

[33] Osterweis M, Solomon F, Green M *et al*. 1984 Monkeys’ responses to separation and loss. In Bereavement:reactions, consequences, and care. Washington, DC: National Academies Press (US). 25032459

[34] McKinney WT. 1986 Primate separation studies: relevance to bereavement. Psychiatr. Ann. **16**, 281–287. (10.3928/0048-5713-19860501-07)

[35] Bloomsmith MA, Tarou LR, Lambeth SP, Haberstroh MD. 2003 Maternal response to mother–offspring separation in the chimpanzee (Pan troglodytes). Anim. Welf. **12**, 359–368. (10.1017/S0962728600025860)

[36] Champoux M, Suomi SJ. 1994 Behavioral and adrenocortical responses of rhesus macaque mothers to infant separation in an unfamiliar environment. Primates **35**, 191–202. (10.1007/bf02382054)

[37] van Oosten JCP, Ploeger A, Sterck EHM. 2025 Recognising depression in non-human primates: a narrative review of reported signs of depression. PeerJ **13**, e18766. (10.7717/peerj.18766) 39802190 PMC11720972

[38] Harlow HF, Suomi SJ. 1974 Induced depression in monkeys. Behav. Biol. **12**, 273–296. (10.1016/s0091-6773(74)91475-8) 4475586

[39] Dettmer AM, Slonecker EM, Clouse S, Ozturkoglu Y, Meyer JS. 2024 No effect of infant nursery rearing on laboratory rhesus monkey dams’ social behavior or long-term cortisol profiles. Appl. Anim. Behav. Sci. **280**, 106428. (10.1016/j.applanim.2024.106428) 39650804 PMC11619073

[40] Meagher RK, Beaver A, Weary DM, von Keyserlingk MAG. 2019 Invited review: a systematic review of the effects of prolonged cow–calf contact on behavior, welfare, and productivity. J. Dairy Sci. **102**, 5765–5783. (10.3168/jds.2018-16021) 31103300

[41] Maestripieri D. 2001 Is there mother–infant bonding in primates? Dev. Rev. **21**, 93–120. (10.1006/drev.2000.0522)

[42] Fernández-Fueyo E, Sugiyama Y, Matsui T, Carter AJ. 2021 Why do some primate mothers carry their infant’s corpse? A cross-species comparative study. Proc. R. Soc. B **288**, 20210590. (10.1098/rspb.2021.0590) PMC8441129 34521250

[43] Klass D. 1999 Developing a cross-cultural model of grief: the state of the field. OMEGA J. Death Dying **39**, 153–178.

[44] Johnson E, Talyigás F, Carter AJ. 2024 Data from: Macaque mothers’ responses to their infants. Zenodo. (10.5281/zenodo.10800611) PMC12000825 40235305

[45] Johnson EA, Talyigás F, Carter A. 2025 Supplementary material from: Macaque mothers’ responses to the deaths of their infants. Figshare. (10.6084/m9.figshare.c.7735659) PMC12000825 40235305

